# Notable mixed substrate fermentation by native *Kodamaea ohmeri* strains isolated from *Lagenaria siceraria* flowers and ethanol production on paddy straw hydrolysates

**DOI:** 10.1186/s13065-018-0375-8

**Published:** 2018-02-05

**Authors:** Shalley Sharma, Anju Arora, Pankhuri Sharma, Surender Singh, Lata Nain, Debarati Paul

**Affiliations:** 1 Division of Microbiology, ICAR-Indian Agricultural Research Institute, New Delhi, 110012 India; 2 Amity Institute of Biotechnology, Amity University, Noida, U.P. India

**Keywords:** Yeast, *Kodamaea ohmeri*, Fermentation efficiency, Mixed sugar fermentation, Inhibitors, Rice straw hydrolysates

## Abstract

**Background:**

Bioethanol obtained by fermenting cellulosic fraction of biomass holds promise for blending in petroleum. Cellulose hydrolysis yields glucose while hemicellulose hydrolysis predominantly yields xylose. Economic feasibility of bioethanol depends on complete utilization of biomass carbohydrates and an efficient co-fermenting organism is a prerequisite. While hexose fermentation capability of *Saccharomyces cerevisiae* is a boon, however, its inability to ferment pentose is a setback.

**Results:**

Two xylose fermenting *Kodamaea ohmeri* strains were isolated from *Lagenaria siceraria* flowers through enrichment on xylose. They showed 61% glucose fermentation efficiency in fortified medium. Medium engineering with 0.1% yeast extract and peptone, stimulated co-fermentation potential of both strains yielding maximum ethanol 0.25 g g^−1^ on mixed sugars with ~ 50% fermentation efficiency. Strains were tolerant to inhibitors like 5-hydroxymethyl furfural, furfural and acetic acid. Both *K. ohmeri* strains grew well on biologically pretreated rice straw hydrolysates and produced ethanol.

**Conclusions:**

This is the first report of native *Kodamaea* sp. exhibiting notable mixed substrate utilization and ethanol fermentation. *K. ohmeri* strains showed relevant traits like utilizing and co-fermenting mixed sugars, exhibiting excellent growth, inhibitor tolerance, and ethanol production on rice straw hydrolysates.
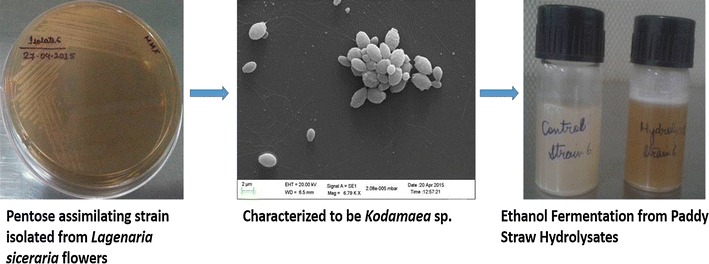

**Electronic supplementary material:**

The online version of this article (10.1186/s13065-018-0375-8) contains supplementary material, which is available to authorized users.

## Background

Recent environmental disturbances, fluctuating prices, and uncertainties associated with the use of conventional fuels, have led to paradigm shift to displace conventional fuels with sustainable, renewable, and environmentally friendly/clean energy sources, among which biomass-derived energy appears to be the most promising option [1]. Of various alternative energy sources, bioenergy derived from lignocellulosic biomass has attracted significant attention as one of the routes to address energy crisis, especially bioethanol in transport sector [2]. Second generation bioethanol, produced by fermenting sugar slurries obtained from enzymatic hydrolysis of cellulose present in lignocellulosic biomass, has the potential of being a major contributor to meet the global energy demand, as biomass is the most abundant, sustainable, and renewable resource on earth. However, unfavorable economics is the foremost impediment in successful deployment of this process on industrial scale. An efficient pretreatment with lower inhibitor generation followed by enzymatic hydrolysis for maximum sugar recovery, and complete utilization and fermentation of all the sugars present in hydrolysates will aid in making the process cost effective [3]. In addition to cellulose, biomass also has hemicellulose, which is the second major polysaccharide, consisting of hexoses and pentoses, with xylose as the major pentose sugar.

Thus, complete conversion of lignocellulosic biomass entails a co-fermenting yeast, capable of fermenting both glucose and xylose yielding high ethanol titers. Development of strains for use in industrial-scale facilities is continuously being carried out in parallel with the process optimization. Commercial strains of *S. cerevisiae,* the most widely used organisms for ethanol production are exclusively involved in glucose fermentation, thus completely utilizing cellulosic fraction while xylose is left unfermented. To overcome this drawback of *S. cerevisiae*, recombinant strains capable of utilizing xylose have been developed since 1980s but ethanol yield was found to be low [4]. Since then, several genetic engineering approaches have been adopted for developing a recombinant strain capable of mixed substrate fermentation but with limited success [5]. This is due to the constraints associated with co-fermentation, like aerobic process of xylose fermentation, co-factor (NADH) imbalance [6] and glucose repression [7, 8]. In addition, inhibitors present in biomass hydrolysates [9] and medium constituents [10] have been observed to affect yeast physiology and fermentation efficiency [11, 12]. All these issues need to be addressed earnestly.

On the other hand, native pentose fermenting yeasts are well known [4, 13]. First report of ethanol production from xylose by yeast came in 1958 when Karczewska [14] observed ethanol production from *Candida tropicalis*. *Pichia* and *Scheffersomyces* are the most interesting pentose fermenting yeasts but their co-fermenting abilities on mixed substrates are yet to be established to the extent suitable for commercial application [15]. Numerous native yeasts are known for xylose assimilation but very few are reported for efficient fermentation of xylose to ethanol. Such yeast include *Pichia, Candida, Pachysolen, Clavispora, Debaromyces, Kluyveromyces, Cryptococcus, Rhodotorula* etc. Researchers have demonstrated low to high ethanol production from xylose in rich medium, by different yeasts isolated from natural habitats like tree bark, decaying wood samples and insect gut [16–18]. Mixed substrate utilization and co-fermentation is still a challenge. Thus, rational bio prospecting for native pentose assimilating and fermenting yeasts is the contemporary approach and increasing efforts have recently been put into evaluating natural xylose fermenting potential of yeasts [19, 20].

A yeast genus *Kodamaea,* earlier placed under *Pichia* genus has been reported for pentose utilization including xylose and arabinose but fermentation of pentoses to ethanol has not been reported. A novel sp. of *Kodamaea*, *K. kakuduensis*, isolated from Australian Hibiscus flower, was reported to be a good glucose fermenter with weak xylose assimilation properties [21]. *Kodamaea ohmeri* has been explored for its food fermentation properties especially for pickling and cocoa beans but ethanol production has not been reported yet [22]. Zhu et al. [23] described d-arabitol as the main product from glucose by *K. ohmeri.* This study illustrates mixed sugar utilization, ethanol fermentation potential, and inhibitor tolerance of two native *K. ohmeri* strains isolated from the flowers of *L. siceraria* plant for their possible exploitation in bioethanol production.

## Experimental

### Isolation of yeast strains

*Lagenaria siceraria* flowers were collected, washed with distilled water and crushed in pestle mortar with 0.8% saline under aseptic conditions. 1 mL of this suspension was inoculated into 50 mL MXYP broth (0.5% malt extract, 1% xylose, 0.5% yeast extract and 0.3% peptone, pH 5) in 100 mL flasks with 0.25% sodium propionate, for enrichment of xylose utilizing yeasts. After 48 h incubation at 30 °C, culture samples were plated on MXYP agar with chloramphenicol (50 µg mL^−1^) antibiotic. Plates were incubated for 24 h at 30 °C and colonies were selected based on their morphology. Selected colonies were purified and grown on same medium and glycerol stocks were prepared.

### Identification and characterization of selected yeast strains

Two potent xylose assimilating strains were selected, strain 5 and strain 6. Both the strains were characterized on morphological, biochemical as well as on molecular level. Phenotypic characterization was done on the basis of their colony and cell morphology using phase contrast microscopy and scanning electron microscopy. Molecular characterization included sequencing of the ITS region of the yeast strains.

### Studying cell morphology using phase contrast microscopy and scanning electron microscopy

To study morphology, overnight grown cultures were observed under phase contrast microscope (Olympus America Inc.) at magnification 10× and 40×. Cell morphology was also studied using scanning electron microscope (Zeiss EVOMA10). Overnight incubated cultures on xylose (1 mL) were centrifuged at 8000*g* for 10 min, 2.5% glutaraldehyde fixative was added to the pellet and kept for 2–4 h to arrest growth. Cultures were then washed with 0.1 M phosphate buffer thrice at an interval of 15 min. Samples were dehydrated with a graded series of acetone (30, 50, 70, 80, 90, 95 and 100%), fixed on cover slips placed over stuff grids. A drop of hexamethyl disilazone was added over the cover slips and then allowed to dry in a fume hood. Cells were observed with scanning electron microscope at an acceleration voltage of 20 kV and images recorded.

### Molecular identification through ITS sequencing

Further confirmation was done by PCR amplification of ITS region. PCR procedures involved denaturation at 95 °C for 5 min, followed by 35 cycles of 94 °C for 5 min, 55 °C for 30 s and extension at 72 °C for 45 s, with final extension for 10 min at 72 °C. Amplified products were run over 1% agarose gel to confirm their molecular size. ITS sequencing of the amplified products was completed by Xcelris, India and further analyzed using Basic Local Alignment Search Tool (BLAST) [24]. Partial sequencing of the strains was done using ITS 1 and ITS 4 degenerate primers i.e., ITS1-forward primer (5′-TCCGTAGGTGAACCTGCGG-3′) and ITS4-reverse primer (5′-TCCTCCGCTTATTGATATGC-3′) [25].

### Biochemical characterization

Ability of *Kodamaea ohmeri* strains to assimilate different sugars was tested using biochemical strips (Hi Media) for yeast. Overnight cultures were inoculated on the strips (100 µL each) and incubated at 28 °C. Results were observed for 72 h.

### Determining enzyme activities

*K. ohmeri* strains were grown for 48 h on 2% xylose, and mixed sugars (2% xylose + 2% glucose) in minimal medium with shaking at 150 rpm at 30 °C. After 48 h, cultures were centrifuged at 8000 rpm for 10 min and supernatant was discarded. Pellet was processed for xylose reductase (XR) and xylitol dehydrogenase (XDH) activities were measured and expressed as specific activities. Protein concentration in crude extracts was measured using BSA as standard.

### Xylose reductase

Pellet obtained was washed twice with phosphate buffer (250 mM, pH 7.0), sonicated, and the lysate was then used as the crude enzyme extract. Two cocktails were prepared as shown in Table 1. Crude enzyme was added to the experimental vial (50 µL) and readings were taken at 340 nm for 3 min and the rate of change of OD was used to determine the activity of the enzyme.

**Table 1:** Reaction cocktail for xylose reductase activity

Solution	Volume (µL)	
	Control	Experimental
DI water	200	100
Potassium phosphate	600	600
2-Mercaptoethanol	100	100
NADPH	50	50
Xylose	0	100

### Xylitol dehydrogenase

Pellet obtained was washed twice with Tris–Cl buffer (500 mM, pH 8.6), sonicated and the lysate was then used as the crude enzyme extract. For this assay, two cocktails were prepared as shown in Table 2, in two separate cuvettes and kept on ice. Crude enzyme (50 µL) was added to the experimental vial and measurements of the rate of change of absorbance per min at 340 nm was measured and considered as the XDH activity for *K. ohmeri* strain 5 and strain 6.

**Table 2:** Reaction cocktail for xylitol dehydrogenase activity

Solution	Volume (µL)	
	Control	Experimental
DI water	300	200
500 mM tris–HCl	400	400
2-Mercaptoethanol	100	100
NAD^+^	100	100
Xylitol	0	100

### Fermentation abilities of *K. ohmeri* strains

Both strains were grown in minimal medium (1.36 mg L^−1^ KH_2_PO_4_, 0.2 g L^−1^ MgSO_4_·7H_2_O, 2.0 g L^−1^ NaCl, 1.0 g L^−1^ (NH_4_)_2_SO_4_, 10 mg L^−1^ FeSO_4_, pH 5) with 5% xylose/10% glucose or both as carbon source for 72 h at 30 °C to check their ability to grow and ferment xylose. Effect of salts like NaCl and FeSO_4_ was studied. Medium (50 mL) in 100 mL Erlenmeyer flasks was inoculated (10% inoculum) and incubated for 72 h at 30 °C. Inoculum was prepared in MXYP broth (pH 7.0) by incubating it at 30 °C for 48 h and shaking (150 rpm). Aliquots were aseptically withdrawn at regular intervals and the absorbance read at 660 nm (Specord 200) to measure growth. These aliquots were then centrifuged at 10,000 rpm for 10 min and supernatants were used for estimation of sugar consumption and ethanol production by HPLC as described later.

### Fermentation of mixed sugars

Cultures were grown on mixed sugars (5% glucose + 5% xylose) as carbon source in minimal medium (10 g L^−1^ KH_2_PO_4_, 5 g L^−1^ (NH_4_)_2_SO_4_, 5 g L^−1^ MgSO_4_·7H_2_O, 1 g L^−1^ yeast extract, pH 5). Composition of minimal medium in this case differed from the above experiment as effect of salts and yeast extract was being monitored. Fermentation was carried out in two phases. Incubation at 30 °C under shaking for 48 h for biomass production was switched to static conditions for ethanol production. Samples were analyzed for growth, sugar consumption and ethanol production. Fermentation efficiency was calculated as [26].

1 $$\begin{aligned} \% Fermentation\;Efficiency \, = \, \left( {Actual\;Ethanol\;Yield \, in \, grams/ \, Theoretical \, Ethanol \, Yield \, in \, grams} \right) \, \hfill \\ \times \, 100 \hfill \\ \end{aligned}$$

2 $$Theoretical \, Ethanol \, yield \, = \, \left( {sugar \, consumed \, in \, grams \, \times \, 0.511} \right)$$

### Stimulation of fermentation ability upon medium supplementation

Effect of medium supplementation with yeast extract and peptone on ethanol production was studied. Treatments with combinations of yeast extract (0.1–1%) and peptone (0.1 and 1%) with pure or mixed sugars (10% glucose or 10% glucose + 5% xylose) were applied. Incubation was carried out as described earlier and samples were analyzed for growth and fermentation.

### Analytical methods

Ethanol levels were estimated using chromatographic techniques, such as HPLC and GC.

### High performance liquid chromatography

Cultures were harvested at regular intervals, centrifuged at 8000 rpm for 10 min, filtered using 0.22 µ syringe filters and subjected to analysis by HPLC. Samples were run on Aminex HPX-87H column (Bio-Rad, Hercules, CA, USA) at 65 °C using 5 mM H_2_SO_4_ as mobile phase at 0.5 mL min^−1^ and measured with a Shodex RI-101 refraction index detector (Shoko Scientific Co. Ltd., Yokohama, Japan). Ethanol concentration and sugar consumption were determined.

### Inhibitor tolerance of *K. ohmeri* strains

For exploitation of *K. ohmeri strains* for fermentation of biomass hydrolysates, it is important to check their capability to grow in presence of HMF, furfural, formic acid and acetic acid, the predominant by-products of biomass pretreatment which are present in hydrolysates and reported to inhibit growth.

Cultures were grown in presence of HMF (0.5–5.0 g L^−1^) and furfural (0.25–0.65 g L^−1^) in minimal medium with 5% glucose + 2.5% xylose and 0.1% yeast extract for 96 h. Growth was checked every 24 h by reading absorbance at 660 nm. Appropriate controls were maintained and growth was compared. Similar experiment was carried out using acetic acid (5–15 g L^−1^) and formic acid (3–11 g L^−1^) under similar conditions. All the experiments were carried out in triplicates.

### Growth and fermentation on biologically pretreated paddy straw hydrolysates

Rice straw of the aromatic rice (Pusa 2511) was pretreated under solid state fermentation using *Trametes hirsute,* for 7 days and cellulose content was analysed in pretreated solids [27]. Enzymatic hydrolysis of biologically pretreated solids was carried out using accellerase^®^1500 (Genencor) loading corresponding to 0.5 mL (~ 15 FPU) per g glucan [28]. Total sugars in hydrolysates were estimated using DNS [29].

Both strains were grown in hydrolysates [30] and culture samples were periodically withdrawn. Samples were processed. Growth and sugar consumption were observed. Ethanol production was detected by HPLC. Defined medium with 1.3% glucose served as control.

Statistical analyses of the results was done using SPSS (Version 21.0. Armonk, NY: IBM Corp).

## Results and discussion

### Growth and characterization

**Fig. 1:**
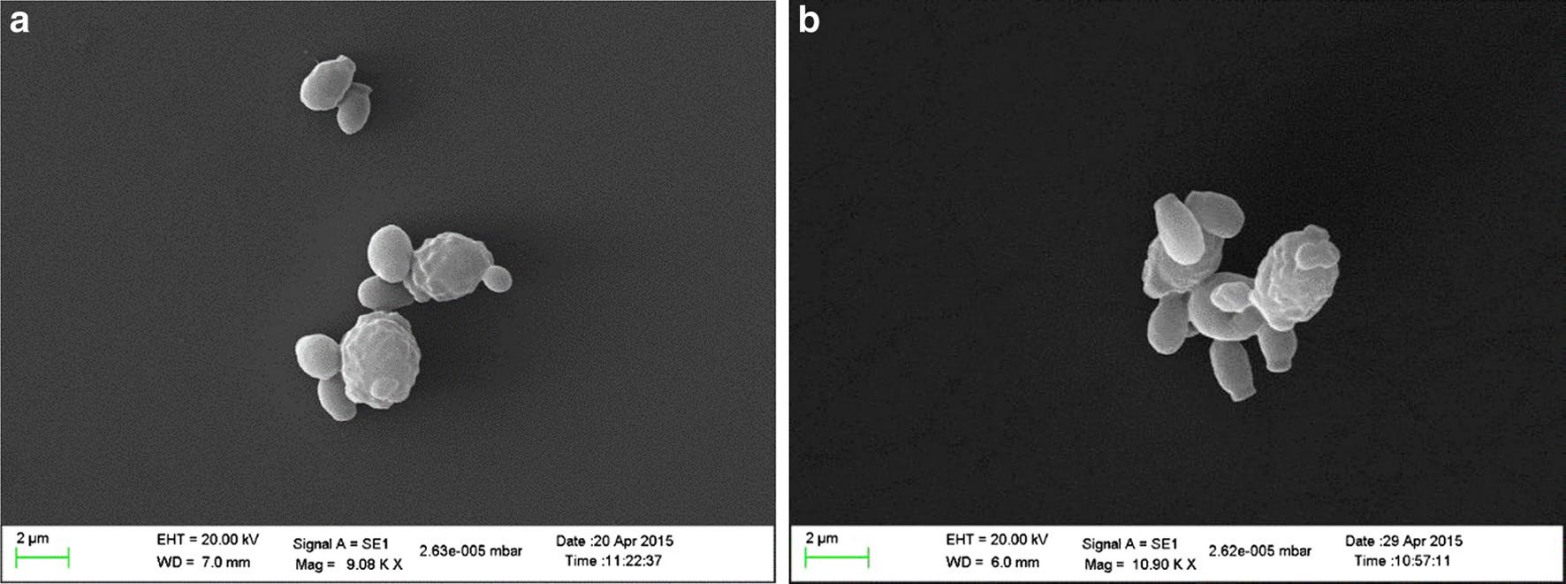
Scanning electron micrographs of strain 5 and strain 6. Cells of strain 5 (**a**) and strain 6 (**b**) appear stressed due to growth on xylose under micro-aerophilic conditions. Budding cells are clearly visible in the electron micrographs

*Lagenaria siceraria* flowers are rich in pentose and hexose sugars and thus used as a source for isolating pentose assimilating *K. ohmeri* strains [31]. *K. ohmeri* strains were isolated and purified from *L. siceraria* flowers by enrichment on MXYP medium and maintained as glycerol stocks. Both the strains grew well on minimal medium with xylose as sole carbon source (Additional file 1: Figure S1). They showed distinct opaque, butyroid, creamy, circular colony morphology with regular margins and raised elevation. Under phase contrast microscope, cells appeared ovoid and occurred singly (Additional file 1: Figure S2). Scanning electron microscopy images showed shrunk cells with irregular margins indicating stress. Budding cells were also observed under scanning microscopy (Fig. 1).

**Fig. 2:**
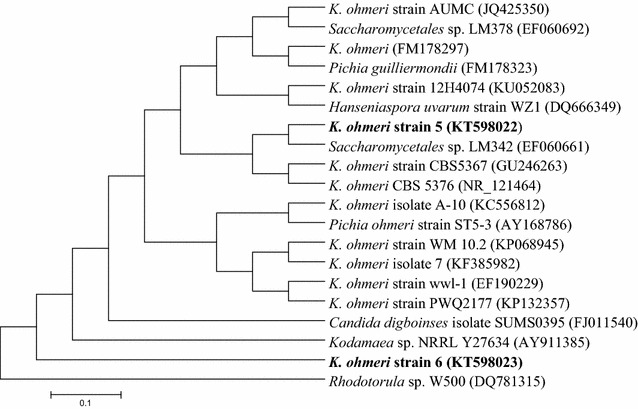
Phylogenetic tree of *K. ohmeri* strain 5 and strain 6

Biochemical characterization showed that both strains could assimilate maltose, sucrose, galactose, cellobiose, raffinose, trehalose, glucose and xylose while inositol, dulcitol, lactose, melibiose were not assimilated and urease test was also negative (Additional file 1: Table S1). Both strains were identified to be *K. ohmeri* upon partial sequencing. Strain 5 (GenBank Accession No. KT598022) showed 100% similarity with *K. ohmeri* while strain 6 (GenBank Accession No. KT598023) displayed 97% similarity. Phylogenetic tree constructed using Maximum-Likelihood [32] also showed their relationship with *K. ohmeri* (Fig. 2). *Kodamaea genus* was earlier placed under *Pichia* genus but was separated later due to considerable genetic distances as measured by partial sequences of 18S and 26S ribosomal RNA and only seven species were placed under the genus *Kodamaea* including *K. anthophila*, *K. kakaduensis*, *K. ohmeri*, *K. laetipori*, *K. nitidulidarum, K. transpacifica, K. meredithae* have been described [33–35].

### Attributes pertaining xylose metabolism

Xylose reductase and xylitol dehydrogenase enzyme activities pertaining to xylose metabolism [36, 37] were exhibited by both the strains but levels were low. The activities suggested the presence of xylose metabolizing pathway in these strains but levels were too low and their ratio predicted the flow of the pathway towards ethanol production. Specific activities (U mg^−1^ protein) of the strains were found to be 0.024, 0.2 (XR) for strain 5 and 6 respectively, while 0.011 and 0.015 (XDH) for strain 5 and strain 6 respectively.

### Fermentation and co-fermentation capabilities and effect of supplementation

**Fig. 3:**
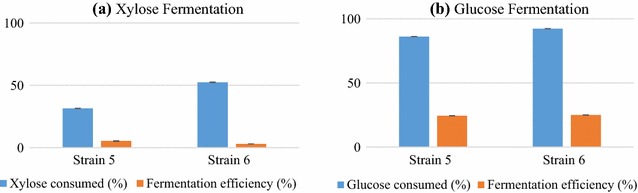
Xylose (**a**) and glucose (**b**) fermentation efficiency on minimal media with salts. Salts hamper the fermentation process as is visible from the lower fermentation efficiencies

As evident from absorbance at 660 nm, both the strains grew well on minimal medium with xylose as sole carbon source and also on mixture of xylose and glucose and fermented them to ethanol (data not shown). On minimal medium containing salts, ethanol was produced by both strains with fermentation efficiency of ~ 25 and ~ 5% on glucose and xylose respectively (Fig. 3; Additional file 1: Table S2). Ethanol was the major product of glucose and xylose fermentation though trace amounts of xylitol and acetic acid were also detected during mixed sugar fermentation. Higher ethanol yield of 0.31 g g^−1^ from glucose with fermentation efficiency of 61% was obtained when minimal medium w as supplemented with 1% yeast extract (YE) and 1% peptone (Table 3) without salts. In a study, d-arabitol production was observed as main product from glucose as the carbon source on rich medium (with 1% YE and 1% peptone) by *K. ohmeri,* and only trace amounts ethanol were observed. Production levels of polyols as fermentation products, largely depend on factors like proper ratio of nitrogen, carbon sources in the medium, original habitat of the fermenting organism and growth conditions [23]. Presence of salts in growth medium influence physiology, hamper growth and distress fermentation efficiency in yeasts while medium with organic supplements augment ethanol fermentation efficiency.

**Table 3:** Glucose utilization and ethanol yield of strain 5 and strain 6

Treatment	Glucose (g L^−1^)			Ethanol yield (g g^−1^)		
Strain 5						
Time (h)	96	108	120	96	108	120
0.1% yeast extract + 0.1% peptone	90.40 ± 16.6	97.95 ± 1.8	88.90 ± 17.2	0.16 ± 0.04	0.28 ± 0.05	0.20 ± 0.09
0.5% yeast extract	99.97 ± 0.06	100	100	0.16 ± 0.09	0.25 ± 0.06	0.28 ± 0.12
1% yeast extract + 1% peptone	97.74 ± 3.71	99.91 ± 0.16	100	0.13 ± 0.02	0.12 ± 0.02	0.12 ± 0.02
Strain 6						
0.1% yeast extract + 0.1% peptone	100	99.9 ± 0.17	100	0.12	0.14 ± 0.01	0.12 ± 0.02
0.5% yeast extract	99.18 ± 1.42	100	100	0.22 ± 0.05	0.24 ± 0.12	0.13
1% yeast extract + 1% peptone	100	99.83 ± 0.21	100 ± 0.01	0.24 ± 0.14	0.31 ± 0.10	0.20 ± 0.10
SE_m_ (±)	1.61	0.23	1.71	0.02	0.02	0.02
CD @5%	4.46	0.64	4.73	0.05	0.06	0.05

Ethanol yield (g g^−1^) = {concentration of ethanol produced (g L^−1^)/concentration of sugar consumed (g L^−1^)}

*SE*_*m*_ standard error of mean, *CD* critical difference

Supplementation with 0.1% YE and 0.1% peptone enhanced fermentation efficiency (to ~ 50%) (Table 4). Further enhancing level of supplementation in medium with higher concentrations of YE/peptone did not increase fermentation efficiency significantly. Studies have suggested significant role of cultivation media components to provide favorable conditions for growth and product formation [10]. Xylose consumption was also enhanced to ~ 40% during co-fermentation and highest ethanol yield was 0.25 g g^−1^ sugar consumed when *Kodamaea* were grown on 10% total mixed sugars (5% glucose + 5% xylose).

**Table 4:** Effect of supplementation on sugar utilization and ethanol yield of *K. ohmeri* strains

Treatment	Xylose consumed (g L^−1^)			Glucose consumed (g L^−1^)			Ethanol yield (g g^−1^)			Fermentation efficiency (%)		
Strain 5												
Time (h)	96	108	120	96	108	120	96	108	120	96	108	120
0.1% (YE + P)	20.47 ± 1.9	20.4 ± 3.2	16.2 ± 3.05	37.1	37.9	37.9	0.22	0.21 ± 0.017	0.22 ± 0.033	44 ± 3.3	40.8 ± 6.5	43.6 ± 5.9
0.5% (YE)	20.3 ± 2.2	18.8 ± 0.57	14.3 ± 1.9	50	50	50	0.16 ± 0.02	0.17 ± 0.01	0.18 ± 0.01	32 ± 4.5	32.5 ± 1.5	34.9 ± 2.4
1% (YE + P)	17.7 ± 2.8	15 ± 7.4	13.3 ± 0.02	50	50	50	0.21 ± 0.03	0.19 ± 0.07	0.2 ± 0.001	41.3 ± 5.4	37.1 ± 14.6	39.7 ± 0.18
Strain 6												
0.1% (YE + P)	11.7 ± 1.7	13 ± 4.3	14 ± 0.45	49.4	49.3 ± 0.19	50	0.25 ± 0.02	0.19 ± 0.05	0.2 ± 0.001	48.6 ± 3.13	38 ± 8.8	39.7 ± 0.25
0.5% (YE)	15.1 ± 9.13	18 ± 6.15	14.2 ± 3.8	50	50	50	0.2 ± 0.01	0.19 ± 0.06	0.2 ± 0.03	38.7 ± 18.1	38 ± 12.1	39.6 ± 6.2
1% (YE + P)	12.2 ± 0.67	66.6 ± 2.4	14.9 ± 0.12	50	50	50	0.25 ± .007	0.18 ± 0.03	0.18 ± 0.001	49.5 ± 1.5	36 ± 5.3	35.1 ± 0.28
SE_m_ (±)	1.24	1.06	0.50	0.00	0.02	0.00	0.01	0.01	0.01	2.46	2.08	1.03
CD @5%	3.41	2.92	1.38	0.00	0.06	0.00	0.03	0.03	0.01	6.80	5.74	2.85

*YE* *+* *P* yeast extract + peptone

Amongst most of the pentose utilizing yeasts only a few have been reported to produce ethanol as major product from pentose fermentation [38]. A mixed sugar fermenting yeast, *Candida lignohabitans* possessing remarkable capability to ferment both pentoses and hexoses, exhibited highest ethanol yield of 0.2 g g^−1^ on rich medium containing 1% yeast extract and 2% soya peptone with 2–5% carbon sources, while no ethanol was detected on minimal medium without supplementation. This might be due to the lower biomass accumulation on minimal medium [7]. In this study, *K. ohmeri* strain 6 exhibited high ethanol yield during mixed substrate fermentation with minimal supplementation. Insignificant increase in fermentation efficiency upon medium with higher supplementation suggested to avoid excessive nutrient supplementation as it favors biomass production [23]. Table 5 shows ethanol yields of related yeast strains. Zheng et al. [3] observed stimulating effect of supplementation on acetone-butanol fermentation using *Clostridium saccharoperbutylacetonicum* and stated that lower supplementation is cost effective and reduces overall production cost.

**Table 5:** Ethanol yields of pentose fermenting strains

Strain	Fermentable sugar	Ethanol yields (g g^−1^)	Reference
*C. lignohabitans*	Glucose + xylose	0.2	[7]
*K. kakuduensis*	Glucose	Traces	[21]
*K. ohmeri*	Glucose	Traces (by product)	[23]
*K. ohmeri* strain 5	Xylose + glucose	0.28	This study
*K. ohmeri* strain 6	Xylose + glucose	0.31	This study

### Inhibitor tolerance

Lignocellulosic biomass is pretreated to facilitate higher conversion of biomass polysaccharides to fermentable sugars such as glucose, xylose, arabinose etc. This process generates by-products which inhibit growth of microbes and obstruct fermentation process. In general, these inhibitors are classified into four groups including lignin degradation by-products (phenolics), sugar degradation by-products (HMF and furfural), and products derived from the structure of the biomass and heavy metal ions (chromium and nickel) [39]. Effect of most commonly found inhibitors like HMF, furfural, acetic acid and formic acid was determined on growth of *K. ohmeri* strains.

**Fig. 4:**
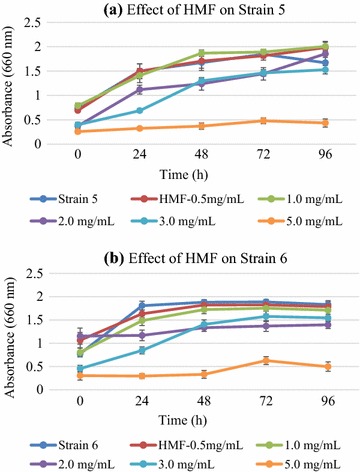
Effect of hydroxy methyl furfural on strain 5 (**a**) and strain 6 (**b**). Growth pattern is similar to the control in case of strain 6 and 0.5–3.0 g L^−1^ concentration of the HMF is not inhibitory for the growth

Concentration ranges were selected based on yields commonly reported in literature and mostly encountered in biomass hydrolysates after different pretreatments [40, 41]. Increasing concentrations of HMF and furfural reduced growth of both strains as compared to controls (Fig. 4). Furfural was inhibitory in initial growth stages but inhibition was gradually overcome upon prolonged growth after 96 h (Additional file 1: Figure S3). This coincided with earlier observations that furfural can reduce growth rate above a certain concentration. It has been proved that furfural inhibits alcohol dehydrogenase (ADH) formation which lead to the accumulation of acetaldehyde intracellularly, causing enhanced lag phase of growth during which enzymes and co-enzymes are produced for the reduction of furfural [42]. HMF also posed similar threats on growth and ethanol productivity of *K. ohmeri* strains as growth was reduced and lesser biomass resulted in lesser ethanol production. *K. ohmeri* strains were found to be tolerant to HMF up to 3 g L^−1^ concentration while at 5 g L^−1^ concentration, growth was reduced.

**Fig. 5:**
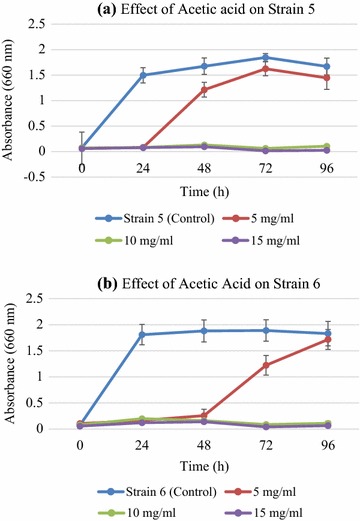
Effect of acetic acid over *K. ohmeri* strain 5 (**a**) and strain 6 (**b**). Strain 6 exhibits a sudden rise in efficiency after 48 h at a concentration of 5 g L^−1^

Effect of organic acids on growth of *K. ohmeri strains* was more pronounced. With formic acid (3–11 g L^−1^) and acetic acid (5–15 g L^−1^), growth was highly affected due to pH change, as optimum pH for yeast growth is 5–6. Formic and acetic acids at concentration used in these experiments reduced pH to 3 leading to reduction in biomass production. In case of acetic acid, there was a sudden rise in growth of both strain 5 and strain 6 after 48 and 72 h respectively. Acetic acid at concentrations up to 6 g L^−1^ did not cause any reduction in growth of the strains [40] (Fig. 5). Acetic acid works by lowering intracellular pH, which is neutralized by plasma membrane’s ATPase by pumping out protons from the cell, thereby, leading to the production of additional ATPs by increasing ethanol production under anaerobic conditions due to enhanced biomass formation. This might be the reason for sudden rise in growth after a certain period as observed in case of *K. ohmeri* strains. Effect of formic acid was more severe and growth of both strains was impeded. Major cause of decreased growth was assumed to be lowering of pH as inhibitory effect of formic acid was nullified when pH was adjusted to optimum (data not shown). This reduction was due to drop in extracellular pH which causes diffusion of undissociated acids inside the cell leading to reduction in intracellular pH [43]. ABE fermentation was repressed by the production of acetic acid produced as a byproduct when *C. saccharoperbutylacetonicum* was grown on eucalyptus hydrolysates [3].

### Growth and ethanol production by *K. ohmeri* strains from biomass hydrolysates

**Fig. 6:**
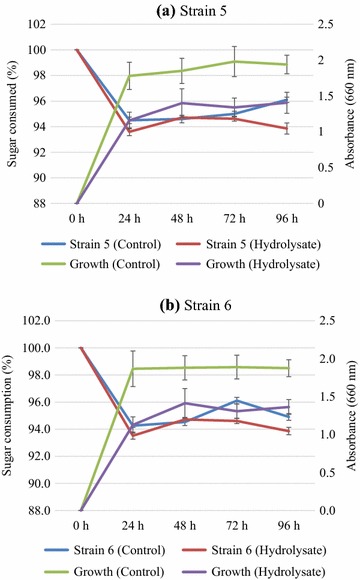
Sugar consumption (%) and growth of *K. ohmeri* strain 5 (**a**) and strain 6 (**b**) on biologically pretreated rice straw hydrolysate

*Kodamaea ohmeri* strains were evaluated for growth and ethanol production on biomass hydrolysates prepared from biologically pretreated rice straw. Total sugar content in the hydrolysates was ~ 1.3% (with 2% glucan loading and 57% saccharification efficiency). Growth on hydrolysates was comparable to the control (Fig. 6). Maximum sugar consumption and ethanol production occurred within 24 h. HPLC analyses of samples showed ethanol production and maximum ethanol level at 72 h by both the strains and it was ~ 2 and 1.3 g L^−1^ by strain 5 and strain 6 respectively (Table 6). Thus, these strains of *K. ohmeri* were able to grow and produce ethanol from paddy straw hydrolysates.

**Table 6:** Ethanol yields of *K. ohmeri* strains from rice straw biomass hydrolysates

Ethanol produced (g L^−1^)	24 h	48 h	72 h	96 h
*K. ohmeri* strain 5 (control)	0.98 ± 0.01	0.065 ± 0.003	0.059 ± 0.0005	0.015 ± 0.0025
*K. ohmeri* strain 5 (hydrolysate)	0.3 ± 0.001	0.35 ± 0.01	1.92 ± 0.04	0.001 ± 0.0015
*K. ohmeri* strain 6 (control)	1.07 ± 0.01	1.15 ± 0.05	0.71 ± 0.02	0.26 ± 0.02
*K. ohmeri* strain 6 (hydrolysate)	0.12 ± 0.03	0.78 ± 0.02	1.28 ± 0.01	0.04 ± 0.008

## Conclusions

Screening for microbes capable of co-fermentation is necessary for efficient conversion of lignocellulosic biomass into ethanol with enhanced productivity. There is a significant advancement in developing a robust microbial strain with co-fermentation potential as well as tolerance to inhibitors. *K. ohmeri* strains, studied here showed promising mixed sugar fermentation potential with enhanced xylose utilization. Strains were also tolerant to HMF, furfural, formic acid and could grow well in presence of acetic acid on prolonged incubation. The study emphasizes that this genus could provide robust native yeast strains with co-fermentation properties which can be evolved further. Lignocellulosic hydrolysates often generate unexpected results due to the presence of inhibitors, as they vary widely in nature [12]. These strains displayed efficient growth and ethanol production from biologically pretreated rice straw hydrolysates.

## Additional file

**Additional file 1. Table S1.** Sugar utilization by *K. ohmeri* strain 5 and strain 6. **Table S2.** Ethanol production, sugar consumption and fermentation efficiency of *K. ohmeri* strain 5 and strain 6 during xylose fermentation. **Figure S1.** Growth of *K. ohmeri* strain 5 and strain 6 on minimal medium with xylose as sole C source. **Figure S2.** *K. ohmeri* strain 5 (**A**) and strain 6 (**B**) as observed under phase contrast microscope. **Figure S3.** Effect of furfural on *K. ohmeri* strain 5 (**A**) and strain 6 (**B**).
