# Supplementary material for: Notable mixed substrate fermentation by native Kodamaea ohmeri strains isolated from Lagenaria siceraria flowers and ethanol production on paddy straw hydrolysates
Source: Chem Cent J. 2018 Feb 5;12:8. doi: 10.1186/s13065-018-0375-8 (PMC5799091; doi:10.1186/s13065-018-0375-8)
Supplement: Supplementary file 1 — Additional file 1. Table S1. Sugar utilization by K. ohmeri strain 5 and strain 6. Table S2. Ethanol production, sugar consumption and fermentation efficiency of K. ohmeri strain 5 and strain 6 during xylose fermentation. Figure S1. Growth of K. ohmeri strain 5 and strain 6 on minimal medium with xylose as sole C source. Figure S2. K. ohmeri strain 5 (A) and strain 6 (B) as observed under phase contrast microscope. Figure S3. Effect of furfural on K. ohmeri strain 5 (A) and strain 6 (B). [file 13065_2018_375_MOESM1_ESM.pdf]

<u>Supplementary Data</u>

Table S1. Biochemical tests for *Kodamaea ohmeri* strain 5 and strain 6

**Biochemical test**	**Strain 5**	**Strain 6**
**Urease**	-	-
**Melibiose**	-	-
**Lactose**	-	-
**Maltose**	+	+
**Sucrose**	+	+
**Galactose**	+	+
**Cellobiose**	+	+
**Inositol**	-	-
**Xylose**	+	+
**Dulcitol**	-	-
**Raffinose**	+	+
**Trehalose**	+	+

Table S2. Xylose fermentation efficiency and ethanol yields of *Kodamaea ohmeri* strain 5 and strain 6

**Strain**	**Concentration of ethanol (mg/mL)**	**Xylose consumed (mg/mL)**	**Theoretical ethanol yield (mg/mL)**	**Fermentation efficiency (%)**	**Ethanol yield g/g sugar consumed**
K. ohmeri **Strain 5**	0.43	15.73	8.02	5.33	0.027
K. ohmeri **Strain 6**	0.41	26.23	13.38	3.05	0.02

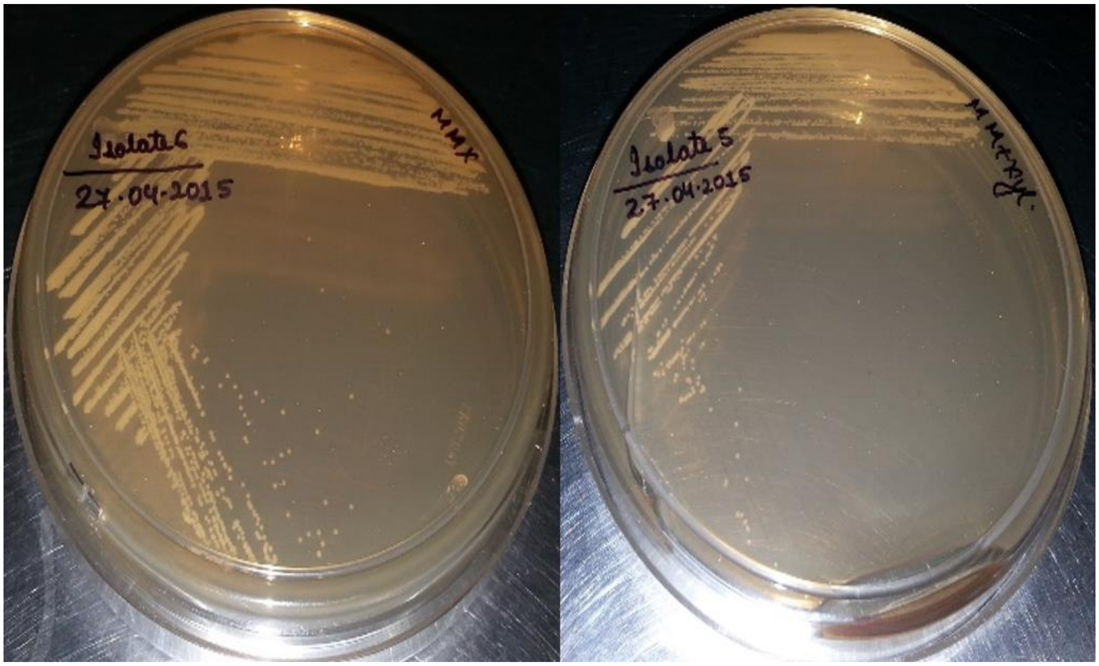

Fig. S1. Colony morphology and xylose assimilation by *Kodamaea ohmeri* strain 5 and strain 6

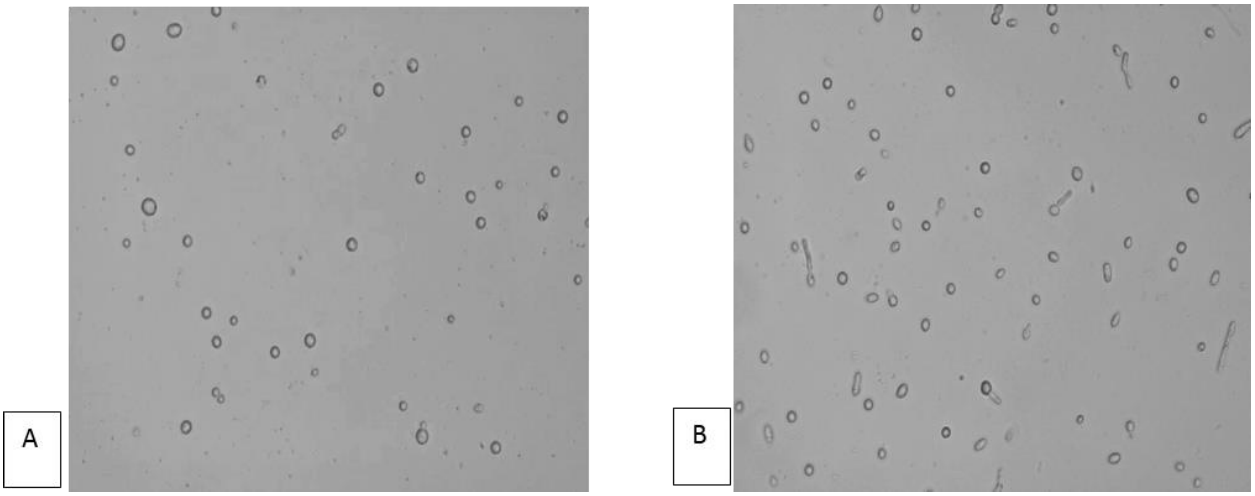

Fig. S2. Cell morphology of *Kodamaea ohmeri* strain 5 (A) and strain 6 (B) as observed under phase contrast microscope

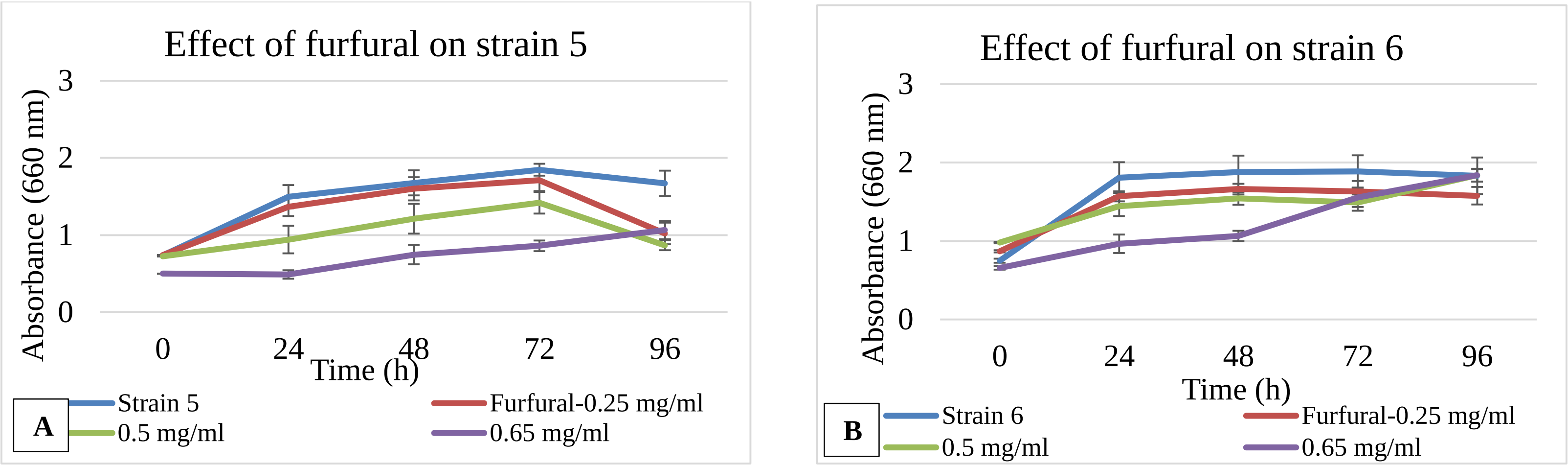

Fig. S3. Effect of Furfural on *Kodamaea ohmeri* strain 5 (A) and strain 6 (B)
